# Rewinding the waves: tracking underwater signals to their source

**DOI:** 10.1038/s41598-017-14177-3

**Published:** 2017-10-24

**Authors:** Usama Kadri, Davide Crivelli, Wade Parsons, Bruce Colbourne, Amanda Ryan

**Affiliations:** 1 Cardiff University, School of Mathematics, Cardiff, CF24 4AG UK; 2 Massachusetts Institute of Technology, Department of Mathematics, Cambridge, 02139 MA USA; 3 Cardiff University, School of Engineering, Cardiff, CF24 3AA UK; 4 Memorial University of Newfoundland, Faculty of Engineering and Applied Science, St. John’s, A1B 3X5 NL, Canada

## Abstract

Analysis of data, recorded on March 8th 2014 at the Comprehensive Nuclear-Test-Ban Treaty Organisation’s hydroacoustic stations off Cape Leeuwin Western Australia, and at Diego Garcia, reveal unique pressure signatures that could be associated with objects impacting at the sea surface, such as falling meteorites, or the missing Malaysian Aeroplane MH370. To examine the recorded signatures, we carried out experiments with spheres impacting at the surface of a water tank, where we observed almost identical pressure signature structures. While the pressure structure is unique to impacting objects, the evolution of the radiated acoustic waves carries information on the source. Employing acoustic–gravity wave theory we present an analytical inverse method to retrieve the impact time and location. The solution was validated using field observations of recent earthquakes, where we were able to calculate the eruption time and location to a satisfactory degree of accuracy. Moreover, numerical validations confirm an error below 0.02% for events at relatively large distances of over 1000 km. The method can be developed to calculate other essential properties such as impact duration and geometry. Besides impacting objects and earthquakes, the method could help in identifying the location of underwater explosions and landslides.

## Introduction

The vast majority of literature on underwater sound (acoustic) waves neglects the effects of gravity, which is justified for many applications because the speed of sound in water far exceeds the maximum phase speed of gravity waves. However, it has been shown recently that gravitational effects play a prominent role under two scenarios.

The first scenario concerns energy exchange between gravity and acoustic waves via resonant triad nonlinear interactions^1,2^. This mechanism may provide a natural explanation of the generation of underwater faint earth tremors, namely microseisms^3,4^, if the elasticity of the sea-floor is considered^5^. Employing a similar mechanism^6^, some of the energy found initially in surface gravity waves can be withdrawn and redistributed over a larger space, resulting in a modulation of the original gravity wave, as argued to be useful by a recent tsunami-induced damage mitigation technique^7^.

The second scenario concerns the propagation of low frequency acoustic waves, e.g. generated from energetic impacts at the surface, underwater explosions, or submarine earthquakes^8^. Here, some of the basic acoustic mode properties, such as amplitude and speed, can be modulated fundamentally^9^. Such modulation proved to be significant, not only when dealing with direct problems, such as the early detection of tsunami^9–11^ or the generation of deep water currents^12^, but also when treating inverse problems^13^.

To properly describe the propagation of acoustic waves under the effects of gravity we employ acoustic-gravity wave theory, which accounts for both the slight compressibility of water and gravitational effects. The propagating acoustic modes are referred to as acoustic–gravity waves, to distinguish them from acoustic waves described using ‘pure’ acoustic theory where gravity is omitted. Although our understanding of underwater acoustic–gravity waves is relatively recent, acoustic–gravity wave theory proved to have a broad spectrum of applications.

In this study, we are concerned with tracking underwater low frequency signals, in particular those generated from impacting objects at the sea surface, originally motivated by locating the missing Malaysian Aeroplane MH370. To this end, we solve the inverse problem which allows calculating the impact time and location. We found that impacting objects have a unique pressure signature, that has been employed to identify a few events, recorded on the Comprehensive Test Ban Treaty Organisation’s (CTBTO) hydrophones, possibly initiated from falling meteorites, and others that could be associated with MH370.

## Results

**Figure 1:**
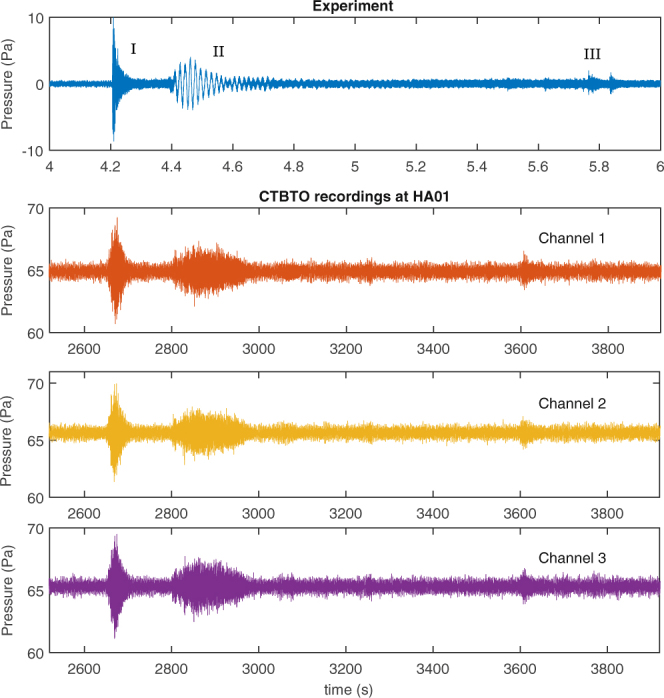
This figure demonstrates the unique structure of an impacting object. Top: signature recorded in experiments of an impacting sphere in a water tank; three spheres were used with diameters = 150 mm, 203 mm, and 305 mm, water depth = 2 m, distance of hydrophone from the impacting object = 20 m; three phases are observed (I) impact, (II) cavitation, (III) reverberation or secondary waves. Bottom three subplots: a signal of similar structure found on HA01 and captured on all three hydrophones, channels 1–3, at 19:45 UTC on March 7th 2014.

To study the pressure signature more carefully, we carried out experiments with weighted spheres that hit the water surface in a water tank (see Methods). The experiments confirmed a unique structure of the signature comprising three regions, I, II, and III, that are believed to be associated with the initial impact, cavitation, and secondary impact or reverberations, respectively (Fig. 1).

### Mathematical model

To evaluate the signature analytically we solve the dynamic problem of a cylinder of radius *R*_*i*_ and height *ζ*_*i*_ penetrating the free-surface vertically downwards during time *τ*_*i*_. The solution of the equation (see Methods) leads to the following farfield bottom pressure:

1 $$\begin{array}{l}{p}_{n}=\tfrac{8\rho c{R}_{i}{\zeta }_{i}}{\pi {r}_{i}{\tau }_{i}}\,\sin \,[\tfrac{(n-\tfrac{1}{2})\,\pi c{\tau }_{i}}{2h\sqrt{1-{({r}_{i}/c\tilde{t})}^{2}}}]\,{J}_{1}\,[\tfrac{(n-\tfrac{1}{2})\,\pi {r}_{i}{R}_{i}}{hct\sqrt{1-{({r}_{i}/c\tilde{t})}^{2}}}]\,\cos \,[\tfrac{(n-\tfrac{1}{2})\,\pi ({r}_{i}^{2}-{c}^{2}{\tilde{t}}^{2})}{hct\sqrt{1-{({r}_{i}/c\tilde{t})}^{2}}}],\end{array}$$

where *ρ* is the density of water, *c* is the speed of sound, *h* is the depth, *r*_*i*_ is the radial coordinate, $\tilde{t}=t-{t}_{i}$ and *J*_1_ is the Bessel function of the first kind, which dominates the envelop of the pressure signal part I in Fig. 1, for the leading mode *n* = 1. The choice of the pressure at the bottom is made for practical consideration as hydrophones are better deployed far from the sea surface and closer to the bottom where the acoustic–gravity wave pressure signature is highest^8^. Note that the farfield, a downward impact or upward elevation result in the same induced pressure field, as the boundary conditions, despite being different, dictate the same mode shape in the farfield. However, the picture is different in the nearfield as the particular solution (surge mode) is different in both cases. Thus, Eq. (1) is in agreement with the farfield pressure relation for an underwater earthquake as given by ref.^13^.

In order to retrieve the impact properties we take an inverse approach which concerns employing the evolution of the signature frequency at given time instants. Solving the boundary-value problem (see Methods), and applying the stationary-phase method a relation of the frequency is obtained in the form:

2 $${\omega }_{n}=\frac{(n-\mathrm{1/2)}\,\pi c/h}{\sqrt{1-{({r}_{i}/c\tilde{t})}^{2}}}.$$

Substituting two frequencies *ω*_*n*_ = (*ω*_*n*,1_, *ω*_*n*,2_), at two time instances *t*_*i*_ = (*t*_*i*,1_, *t*_*i*,2_) in (2) yields a system of two equations with two unknowns, namely *r*_*i*_ and *t*_*i*_. Solving the system, the impact distance and time are obtained. The direction of the impact (bearing) is calculated using an array of nearby hydrophones, if applicable; in this case, only one distance solution from a single hydrophone is required whilst other solutions from additional hydrophones can be employed to address the uncertainties involved, which is a major advantage of the proposed method. The accuracy of the proposed method was also evaluated numerically and showed errors below 0.02% for 1000 ≤ *r* ≤ 10000 km (see Methods).

### Analysis of CTBTO data

**Figure 2:**
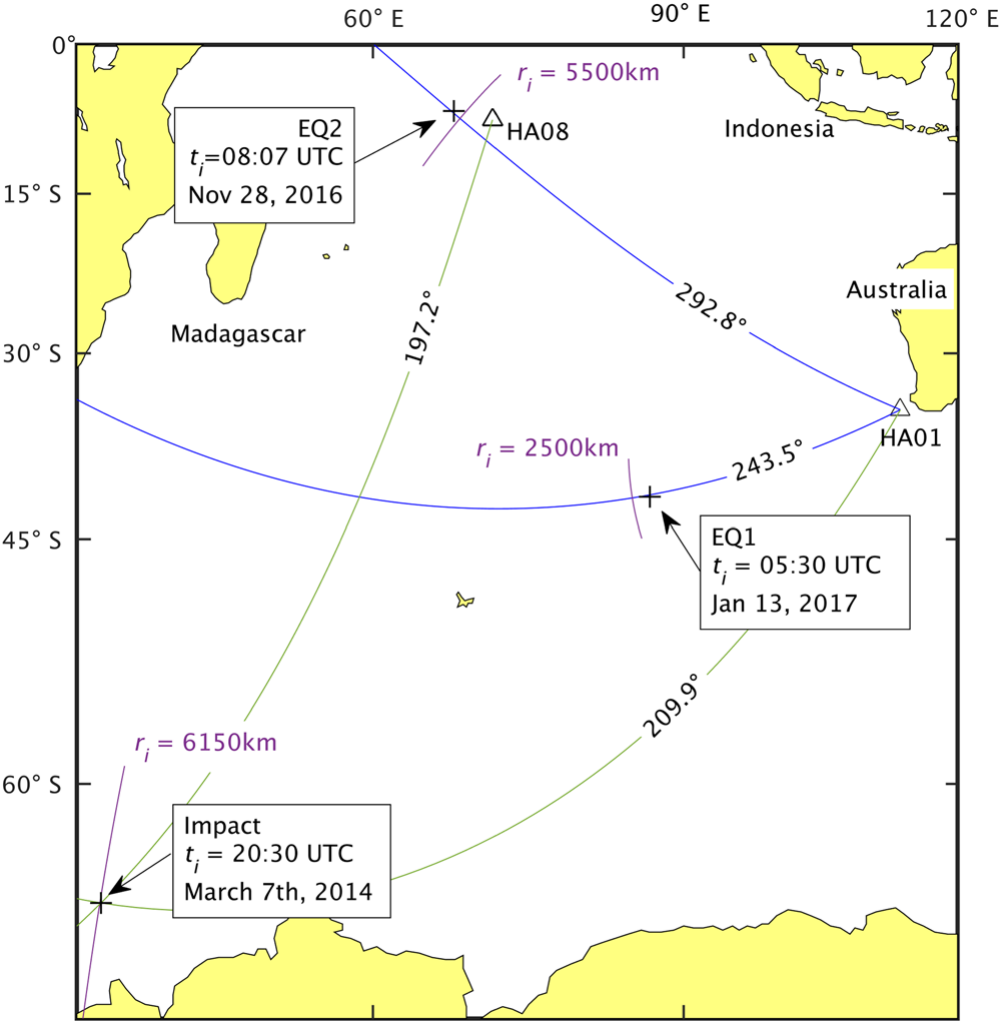
Great-circle orthodromic (or “shortest distance”) arcs (blue) from the hydrophone station HA01 indicate the bearing relative to the station of the incoming acoustic-gravity waves generated by distinct events. The distance *r*_*i*_ (purple arc) from the station is calculated using the inverse problem, and represents the highest probability (mode). The epicentre locations of the two earthquakes EQ1 and EQ2 and the triangulation from the station HA08 and HA01 for the 21:31:24 UTC event (green arcs) are also shown (+), together with the distance calculated with the inverse method. (Generated with Matlab R2016b Mapping Toolbox https://uk.mathworks.com/products/mapping.html. Public domain coastline data derived from Digital Chart of the World (DCW) available online at http://gis-lab.info/qa/vmap0-eng.html).

We have analysed a total of 18 hours of time series data from CTBTO’s hydroacoustic station HA01, comprising of three hydrophone channels (see Methods). The analysis revealed a few strong signals that have almost an identical structure to that found in the experiments (Fig. 1). One of these, received at HA01 at 21:31:24 UTC, could be triangulated with another hydroacoustic station, known as HA08, at −65.5445°, 32.4730°. Using the inverse method, we have identified the location to be at 6250 km from HA01, which closely matches the triangulation result (Fig. 2). The strength and location of the signals suggest an energetic impact that could have been caused by falling meteorites. It is worth mentioning that between 18,000 to 84,000 meteorites bigger than 10 grams fall to Earth annually, and the signals could possibly belong to the larger ones among the falling meteorites^14^. In this regards, the proposed method could be beneficial for meteoric studies.

Since the actual CTBTO data is limited by its sampling frequency (250 Hz), and introduces various levels of signal disturbances, there is a need to validate the method against data from sources with known locations. To this end, we analysed the following recent earthquakes both 5.1 in magnitude and 10 km in depth:

1. EQ1: occurred on 05:30 Jan 13, 2017 UTC; epicentre at −6.762°, 68.546°.
2. EQ2: occurred on 08:07 Nov 28, 2016 UTC; epicentre at −41.857°, 88.545°.

To each bearing, a clockwise systematic error was added, according to^15^, corresponding to 0.8°. For EQ1 and EQ2, we calculated bearings of 243.5 ± 0.3° and 292.8 ± 0.4° which are in agreement with bearings calculated by standard means as 243.1° and 293.0° relative to HA01, respectively. The distances of EQ1 and EQ2 are 2360 km and 5600 km, as calculated using triangulation from various seismic sensors. Here, one advantage of the proposed method is that we are able to calculate the distance independently using a single hydrophone. In the case of EQ1, the calculated distance is 2500 km as opposed to 2360 km. In the case of EQ2, the calculated distance is 5500 km as opposed to 5600 km.

**Figure 3:**
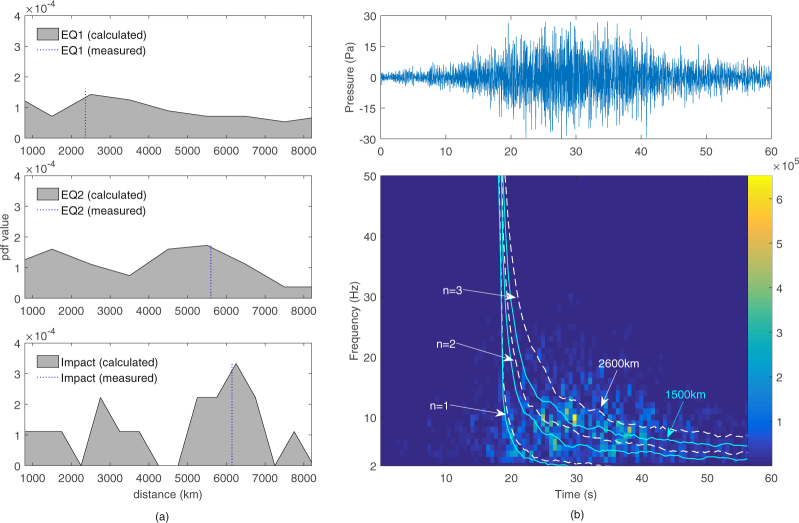
(**a**) Distance probability density function for EQ1, EQ2 and the impact event. These are calculated by using multiple pairs of (*ω*_*n*_, *t*) in order to address noise in the signals. The peaks in the PDF show the highest probability distance from HA01 which closely match the earthquakes location, shown in dashed lines. (**b**) EQ1 signature from the hydrophone’s channel 1 (above) and its time-frequency spectrogram (below). The spectrogram shows the points in the time-frequency plane where most of the energy is contained. By overlaying the direct problem frequency content for modes 1 to 3, the modes are compatible with a source distance between 1500 km (cyan solid lines) and 2600 km (white dashed lines).

We applied the above method to the two earthquakes EQ1 and EQ2 (using epicentre coordinates), and to the impact location (estimated with triangulation from HA01 and HA08). The measured distances from the epicentres are 2360 km and 5600 km for EQ1 and EQ2, and 6145 km for the impact. To address the high noise content of the signals, the inverse method is applied to multiple pairs of instantaneous frequencies and times, in order to extract a probability distribution of distances. The most probable calculated distance, using the inverse method, is 2500 km and 5500 km for EQ1 and EQ2, and 6150 for the impact (Fig. 3a). As a further confirmation we calculated the direct solution for the first three modes using a distance of 1500 km and 2600 km, and we extracted the average frequency evolution in time. We then overlaid the direct solution with the spectrogram of EQ1 to give a satisfactory match (Fig. 3b).

**Figure 4:**
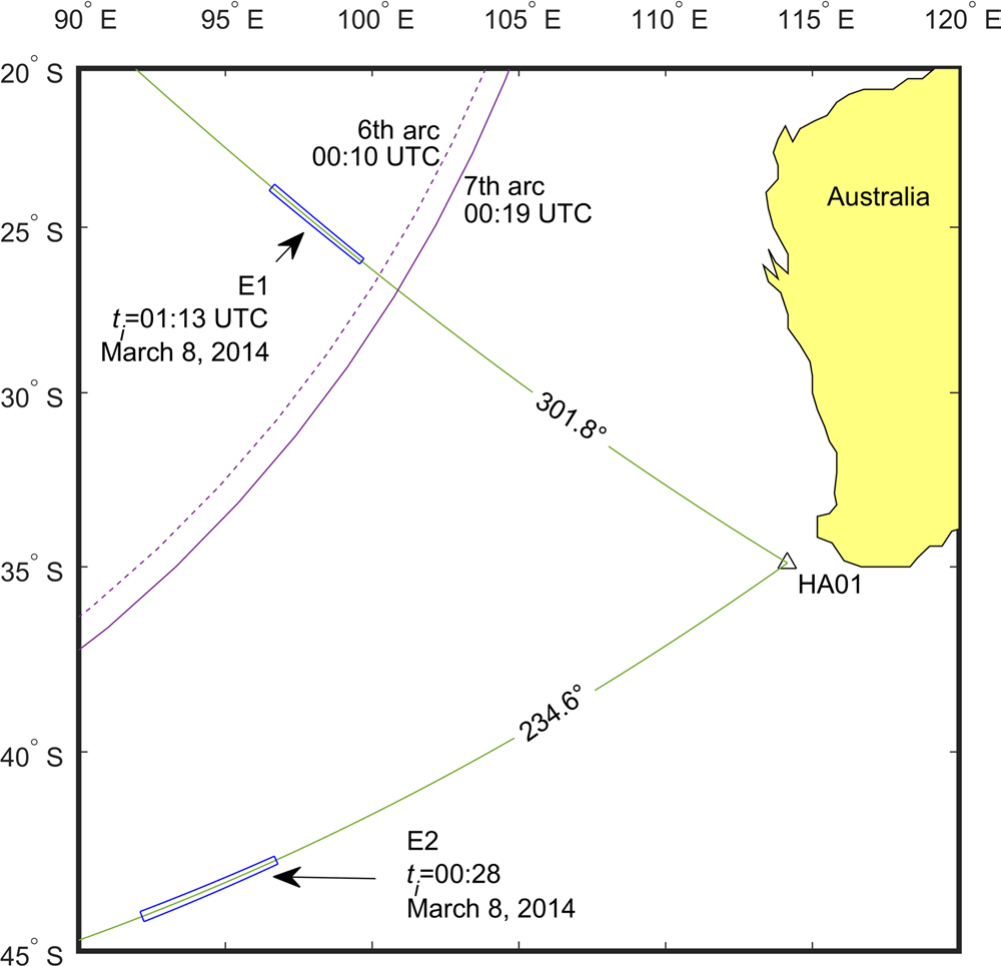
The map shows the two significant events found in CTBTO data from March 8th 2014, between 00:00 UTC and 02:00 UTC. The most probable region of each source is shown in blue rectangles, considering angular and distance uncertainties. For reference, the 6th arc (dashed purple) and 7th arc (solid purple) calculated from the last two MH370 satellite handshakes on March 8th 2014 are shown on the map. (Generated with Matlab R2016b Mapping Toolbox https://uk.mathworks.com/products/mapping.html. Public domain coastline data derived from Digital Chart of the World (DCW) available online at http://gis-lab.info/qa/vmap0-eng.html).

A particular focus of the study was given to data recorded on March 8th 2014 between 00:00 and 02:00 UTC, a time window in which MH370 is believed to have crashed in the Southern Indian Ocean. Only two remarkably weak signals were identified as potentially associated with MH370. The poor quality of the signals resulted in a relatively large uncertainty in the locations (Fig. 4):

1. E1: 301.4 ± 0.4°, 1900 ± 200 km from HA01, centred at −23.662°, 96.676°, recorded at 01:34:40 UTC (event source between 01:11 and 01:16).
2. E2: 234.6 ± 0.4°, 1940 ± 200 km from HA01, centred at −43.487°, 94.469°, recorded at 00:50:00 UTC (event source between 00:25 and 00:31).

The event E2 was recorded only a few minutes after the last transmission time (handshake) between MH370 and the satellite, though over 500 km away from the last handshake, which corresponds to a location on an arc known as “7th arc” (Fig. 4). This would have required MH370 to travel at a speed over 3300 km/hr for 9 minutes, which is unlikely to be possible. Also, note that MH370 is believed to have crashed close to the 7th arc due to fuel considerations.

For the signal arriving from E1 a similar bearing was reported by^16^ as 301.6 ± 0.75°. Here, it is notable that E1 is in a close proximity to the 7th arc, though from west, and two possible scenarios could have occurred after the last handshake while traveling at a standard speed: (1) MH370 travels for a duration of about 50 minutes into zone E1; (2) MH370 travels for a much shorter time, but the signal comes from a delayed implosion or impact with the sea floor. Both scenarios are left for the search experts to discuss.

## Discussion

Acoustic and gravity wave modes are virtually decoupled, and gravitational forces only slightly modify the acoustic properties, in particular when the frequencies of interest are relatively high. Nevertheless, when the frequencies are low, say below 5 Hz, gravitational forces can alter the propagating acoustic mode properties. In addition, even at higher frequencies applying an acoustic–gravity wave approach could be essential for obtaining a proper description for the acoustic mode shapes, which can differ largely if the traditional acoustic approach was employed instead.

Motivated initially by locating the missing Malaysian Aeroplane MH370, we studied the radiation of acoustic–gravity waves from impacting objects. It turns out that while impacts of objects on the sea surface have a unique pressure signature the evolution of acoustic–gravity waves is similar regardless to the generating source as long as the farfield is concerned. Therefore, the proposed method for tracking underwater acoustic–gravity wave signals is relevant to broad applications, from locating falling meteorites to detecting landslides, snowslides, storm surges, and rogue waves. Moreover, implementing a similar approach one might be able to develop similar inverse solutions for a variety of problems of somewhat different nature and disparate scales, such as the detection of failures in aeroplanes, flow configurations in pipelines, and movements in Earth’s interior and plate tectonics, among others. As a final note, it has been now over three years since MH370 has disappeared, with absolutely no clue on its location, despite the large efforts that have been put into the search where any new insight should have been considered. Here, we propose a method that is able to accurately track underwater signals to their source using acoustic–gravity wave theory, provided recordings of high quality and resolution. We hope our paper would encourage experts in data analysis and researchers to refine the available CTBTO data further, which would enhance the location certainty of current and possibly newly identified signals.

## Methods

### Lab experiments

Experiments were conducted to establish the acoustic signature of an object impacting the water surface. The methodology consisted of dropping weighted spheres into a towing tank some distance from an active hydrophone. The hydrophone was located close to the tank bottom and the acoustic signal was recorded for subsequent analysis.

#### Facility and Equipment

**Figure 5:**
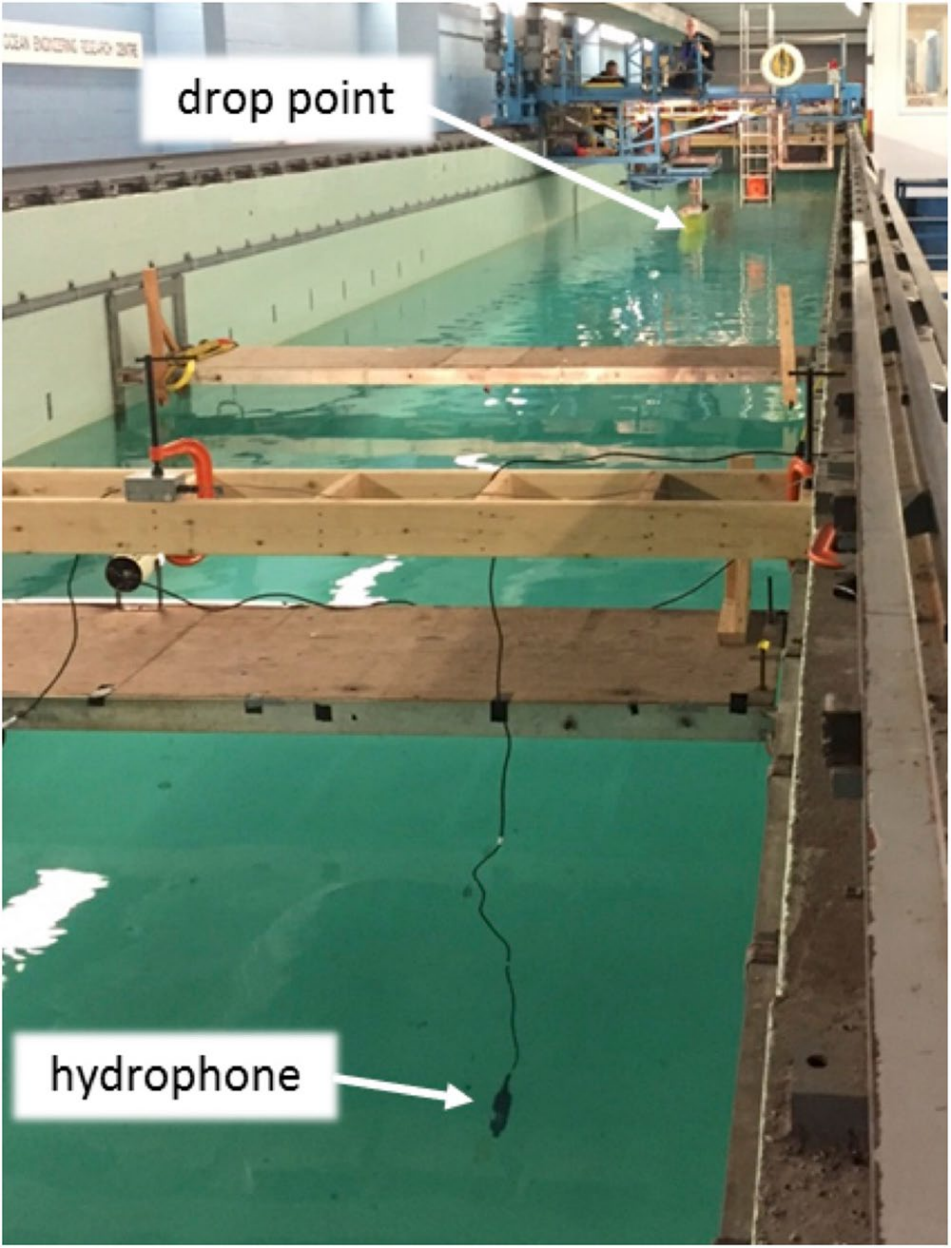
Towing tank at the Faculty of Applied Science and Engineering Towing Tank at Memorial University of Newfoundland (MUN). Three sizes of hollow steel sphere were dropped from the far end of the tank (see white circle) to create an impact at the water surface; sphere diameters 150, 203, and 305 mm, and mass 1.6, 4.0 and 13.4 kg, respectively. The radiated acoustic–gravity waves from the impacting sphere was recorded by a hydrophone type Ocean Sonics icListen HF −200 m model SB2-ETH S/N 1267 (www.oceansonics.com).

The experiments were conducted in the Faculty of Applied Science and Engineering Towing Tank at Memorial University of Newfoundland (MUN) in St. John’s, NL Canada. The tank is 57.4 m long, 4.5 m in width, with a maximum depth of 3.04 m. The tank water depth was at 2.0 m. Figure 5 shows a view of the facility during the experiment.

The hydrophone used for these experiments is an Ocean Sonics icListen HF −200 m model SB2-ETH S/N 1267. This instrument has a stated frequency range of 10 Hz to 200 kHz and a depth rating of 200 m. The Ocean Sonics digital hydrophones do not require preamplifiers, filters or digitisers for signal conditioning but provide output directly as a binary stream. The Smart Hydrophone series add a processor and memory to the digital hydrophone thus allowing data to be processed within the hydrophone unit and then either streamed or recorded in the instrument. The smart hydrophones also carry their own calibration. In the experiments conducted for this study, hydrophone data was streamed directly to a laptop PC. Three sizes of hollow steel sphere were used to create the surface disturbance. The spheres have diameters 150, 203, and 305 mm, and mass 1.6, 4.0 and 13.4 kg, respectively. The spheres are hollow stainless steel fishing floats that have been filled with paraffin wax so that the specific gravity of each sphere is approximately 0.92. This gives each sphere a small positive buoyancy.

**Figure 6:**
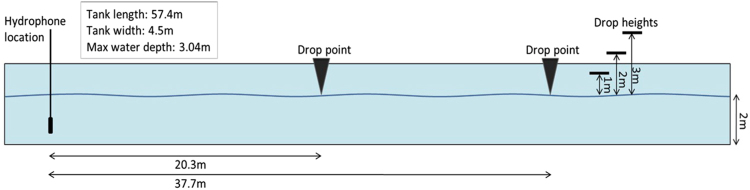
Schematic diagram of experimental setup. A total of 18 tests were performed, one for each sphere, vertical drop height and horizontal distance between drop point and the hydrophone. When the specified horizontal distance and drop height for each individual test was confirmed, the person dropping the sphere signalled to begin the recording. Once data began collecting, the sphere was released. Recording times for each test ranged between 30 s to 1 min, ensuring the full impact was logged. Spheres were released and allowed to fall to the surface, where they impacted and then sank to a distance that was dependent on sphere size and drop height but was not measured.

The hydrophone was located at a depth of 1.7 m below the water surface, which is 0.3 m from the tank bottom, and 1 m from the east wall of the tank, as shown in Fig. 5. Spheres were manually dropped from three heights measured with respect to the water surface. A minimum waiting period of 3 min was allowed between tests. The distance between the hydrophone location and the drop location was measured with a laser distance finder but is estimated to be accurate to within 0.25 m due to the manual nature of the drop. A schematic diagram of the experiment is shown in Fig. 6.

The hydrophone was connected to a laptop previously installed with the Ocean Sonics Lucy Software v4.2.0 for hydrophone data collection. Each recording performed by the Lucy software provided both a.txt text file and a.wav media file for analysis. Prior to completing the experiments, initial recordings were taken of the hydrophone at resting 1.7 m water depth for reference. When the specified horizontal distance and drop height for each individual test was confirmed, the person dropping the sphere signalled to begin the recording. Once data began collecting, the sphere was released. Recording times for each test ranged between 30 s to 60 s, ensuring the full impact was logged. This process was repeated for every test. Spheres were released and allowed to fall to the surface, where they impacted and then sank to a distance that was dependent on sphere size and drop height but was not measured. Approximately 30 s after the sphere had returned to the surface it was retrieved using a light rope that was attached to a screw eye bolted into the sphere.

The test matrix was set to provide a range of impact energy and a range of distances between the source and measurement within the physical dimensions of the tank. The 3 m height was the maximum that could be achieved from the carriage platform and the maximum distance between the hydrophone and drop location was limited by the available length of the tank. Test order was not randomised.

#### Comparison with CTBTO data

**Figure 7:**
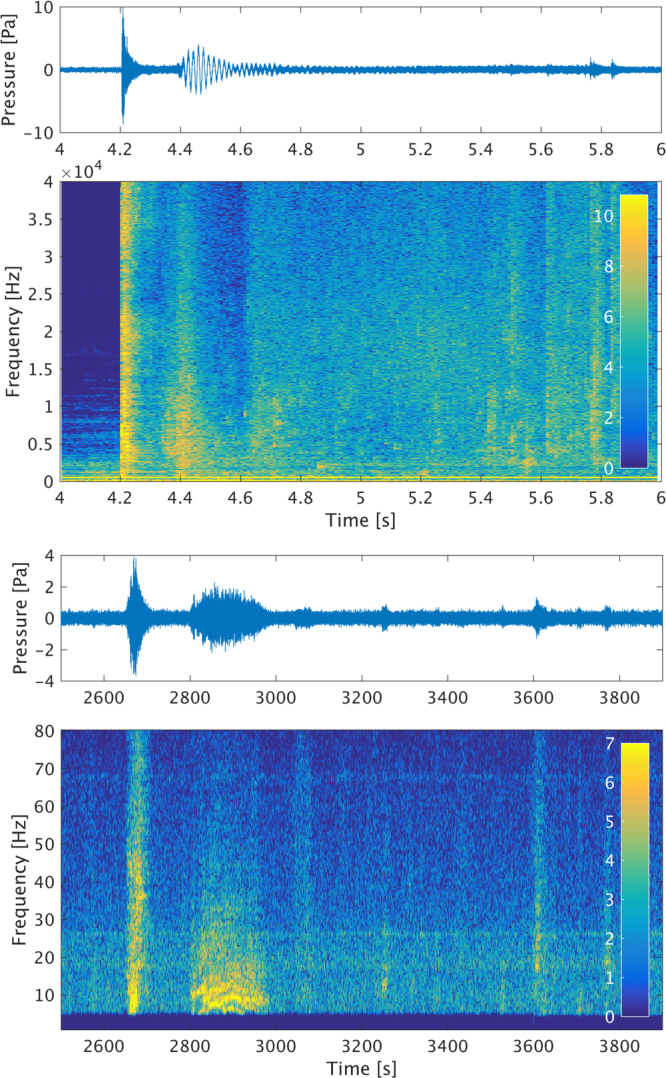
Signals and spectrograms from experiment (top) and measurement at HA01 (bottom). The frequency content and duration in the measured signal are altered and filtered by the varying sea depth and the dispersion that the signal undergoes while traveling for more than 6000 km. In the experiment, the initial phase has a clear impulsive nature, while the second phase appears to contain a mid frequency precursor to the bulk of the signal, which could be due to water starting to fill the air cavity before its collapse. The lower frequencies are masked by the tank resonance, found at around 500 Hz. Some lower amplitude signals, potentially related to tank reflections, can be seen in the spectrogram between 5.4 and 5.6 s. The broadband noise that follows the impact is also consistent with tank reverberation, which is absent before the impact (4 to 4.2 seconds).

The experimental signal has an overall shape that closely resembles the signal identified in the CTBTO data at the HA01 station. The time scale and hence the frequency content are however different due to the effect of the long distance propagation. As frequencies will travel at different velocities due to dispersion, the signal will spread further in time as it travels longer distances. Higher frequencies will attenuate faster, and the effective size of the disturbance will influence the energy distribution at lower modes. Moreover the HA01 array sample rate is limited to 250 Hz, which gives an upper limit for the frequencies at around 100 Hz. This effect is shown in Fig. 7.

### Analytical solution

The governing equation in cylindrical form (*x* ^2^ + *y* ^2^ = *r* ^2^) for linearised, inviscid motion in a compressible medium with velocity determined by the gradient of a potential, *u* = ∇Φ is given by

3 $${{\rm{\Phi }}}_{tt}-{c}^{2}\,({{\rm{\Phi }}}_{rr}+\frac{1}{r}{{\rm{\Phi }}}_{r}+{{\rm{\Phi }}}_{zz})+g{{\rm{\Phi }}}_{z}=0;-h\le z\le 0.$$

The bottom boundary condition is given by

4 $${{\rm{\Phi }}}_{z}=\mathrm{0;}\quad z=-h\mathrm{.}$$

where *g* is gravitational acceleration. The gravitational term *g*Φ_*z*_ has a negligible contribution to the propagating modes^17^ and thus was omitted in the analysis. As such, the gravitational forces come into play only through the boundary conditions. Here, the kinematic and dynamic boundary conditions at the free surface (with impact) are

5 $$\{\begin{array}{l}{\eta }_{t}={{\rm{\Phi }}}_{z};\\ g\eta +{{\rm{\Phi }}}_{t}+\frac{\delta P}{\rho }=\mathrm{0;}\end{array}\quad z=\mathrm{0,}$$

where *η* is the surface displacement, *ρ* is the density of water and *δP* is the pressure exerted by the impacting source on the water column. In the case of a submarine earthquake the bottom boundary condition becomes Φ_*z*_ = *ζ*_*i*_/*τ*_*i*_ whereas *δP* = 0 at the free surface condition. Applying a separation of variables in the linearised field equation results in an ordinary differential equation.

#### Dispersion relation

Defining the Fourier transform of the velocity potential

6 $$\phi (r,z,\omega )=\frac{1}{\sqrt{2\pi }}\,{\int }_{-\infty }^{\infty }\,{\rm{\Phi }}(r,z,t)\,\exp \,(-i\omega t){\rm{d}}t$$

the governing equation and boundary conditions become

7 $$\frac{{\omega }^{2}}{{c}^{2}}\phi +{\nabla }_{r}^{2}\phi +{\phi }_{zz}=0,\quad -h\le z\le 0$$

8 $$-{\omega }^{2}\phi +g{\phi }_{z}-\frac{{\zeta }_{0}}{\tau } {\mathcal H} \,({R}^{2}-{r}^{2})\, {\mathcal H} \,(t(\tau -t))=0$$

9 $${\phi }_{z}=\mathrm{0,}\quad z=-h$$

where ${\nabla }_{r}^{2}$ is the horizontal gradient, and ${\mathcal H}$ is the Heaviside step function. Using the method of separation of variables the field equation results in two ordinary differential equations, which upon substitution in the boundary conditions result in the well known dispersion relation which is needed to calculate the AGW modes,

10 $${\omega }^{2}=-g{\kappa }_{n}\,\tan ({\kappa }_{n}h),\quad {\kappa }_{n}^{2}=\frac{{\omega }^{2}}{{c}^{2}}-{k}_{n}^{2}$$

where subscript *n* denotes the mode number. For AGWs *κ*_*n*_ and *k*_*n*_ are real, representing the vertical and horizontal wavenumbers and *n* = 1 … *N* where *N* is the highest possible AGW number; for the ‘gravity’ mode *κ*_0_ is imaginary and *k*_0_ is real; and for the evanescent modes *k*_*n*_ is imaginary, with *n* > *N*.

#### Farfield bottom pressure

The velocity potential is found by constructing inner (*r* < *R*) and outer (*r* > *R*) regions. For the inner region one needs to include a particular solution,

11 $$s(z)=\frac{i{\zeta }_{0}c}{\tau \sqrt{2\pi }}\frac{1-{{\rm{e}}}^{-i\omega \tau }}{{\omega }^{2}}\frac{\alpha \,\cos \,(\frac{\omega }{c}z)+\,\sin \,(\frac{\omega }{c}z)}{\cos \,(\frac{\omega }{c}h)+\alpha \,\sin \,(\frac{\omega }{c}h)}$$

12 $$\alpha =\frac{g}{\omega \,c}$$

The particular solution represents the surge mode that exists only within the region *r* < *R* where the fluid is suddenly displaced (upwards or downards). For a free surface *α* reduces to *g*/*ωc* and the solution originally obtained by ref.^13^ is retrieved. Following a standard matching technique between the inner and outer regions, and applying continuity at *r* = *R*, we obtain a solution for AGW modes in the outer region of the form

13 $$\begin{array}{rcl}{{\rm{\Phi }}}^{({\rm{out}})}(r,z,t) & = & -\frac{4R{\zeta }_{0}}{\tau }\,\sum _{n=1}^{N}\,{\int }_{{\omega }_{sn}}^{\infty }\,\frac{{\kappa }_{n}}{\omega {k}_{n}}\frac{\cos \,{\kappa }_{n}(z+h)\,\sin \,(\omega \tau \mathrm{/2})}{\sin \,\mathrm{(2}{\kappa }_{n}h)+2{\kappa }_{n}h}\\  &  & \times {J}_{1}({k}_{n}R)\,[{J}_{0}({k}_{n}r)\,\sin \,(\omega t-\frac{\omega \tau }{2})-i{Y}_{0}({k}_{n}r)\,\cos \,(\omega t-\frac{\omega \tau }{2})]\,{\rm{d}}\omega \end{array}$$

where *J* and *Y* are Bessel and functions of the first and second kinds.

The bottom pressure is given by

14 $${p}_{b}=-\rho {{\rm{\Phi }}}_{t},\quad z=-h\mathrm{.}$$

For large distance approximation, substituting (14) into (13) and applying the method of stationary phase the farfield bottom pressure is obtained for the leading AGW modes (see also ref.^13^, eq. (23))

#### Solution for the inverse problem

We seek an approximation for the acoustic–gravity bottom pressure signature at large distances. Define the phase of mode *n* as

15 $${f}_{n}(\tilde{\omega })={k}_{n}\frac{{r}_{i}}{\tilde{t}}-\tilde{\omega },$$

where

16 $${k}_{n}^{2}(\tilde{\omega })=\frac{{\tilde{\omega }}^{2}-{\omega }_{sn}^{2}}{{c}^{2}},$$

and *ω*_*sn*_ can be approximated by^18^:

17 $${\omega }_{sn}=\frac{(n-\mathrm{1/2})\pi c}{h},\quad n=1,2,\ldots \mathrm{.}$$

At the stationary point $\partial {f}_{n}/\partial \tilde{\omega }=0$, so that

18 $$\frac{\partial {f}_{n}}{\partial \tilde{\omega }}=\frac{\tilde{\omega }}{\sqrt{{\tilde{\omega }}^{2}-{\omega }_{sn}^{2}}}\frac{{r}_{i}}{c\tilde{t}}-1=0.$$

At the stationary phase $\tilde{\omega }={\omega }_{n}$, and thus we can finally write

19 $${\omega }_{n}=\frac{(n-\mathrm{1/2)}\,\pi c/h}{\sqrt{1-{({r}_{i}/c\tilde{t})}^{2}}}\mathrm{.}$$

Making use of the CTBTO data, upon substitution of the frequencies of the same hydrophone and the corresponding times, *r*_*i*_ is finally found. Note that since the bearing is known (see bearing calculation below), data from other hydrophones or at other times can be used for uncertainty and sensitivity analysis. If, however, the bearing was not known, one could alternatively repeat the process using another two distant hydrophones all of which result in three different radii *r*_*i*_. The intersection of circles with the calculated radii result in the intersection point (triangulation), i.e. the location of the event. Note that applying a similar approach as given by ref.^13^ one can calculate the size, duration, and effective radius of the impacting object.

#### Numerical validation

To assess the accuracy of the proposed method we performed a numerical validation where input distances were compared to distances calculated by the inverse solution. For a set of radial distances, 5 ≤ *r* ≤ 10000 km, this validation exercise clearly demonstrates the high accuracy of the method, with an error below 0.02% for events at relatively large distances of over 1000 km, as summarised in Table 1. Note that the method uses large distance approximation, and thus its effectiveness reduces for relatively short distances, e.g. below 5 km, where the error becomes remarkably large.

**Table 1:** Numerical validation at various distances.

Distance (km)		% Error
Actual	Calculated	
5.000	4.185	16.3000
50.000	55.138	10.2760
500.000	498.162	0.3676
1000.000	1000.163	0.0163
5000.000	5000.681	0.0136
10000.000	10000.266	0.0027

### Signal processing and bearing calculation

The hydrophones at HA01 are arranged in a triangular shape spaced by around 2000 m and located at the following coordinates:

- Channel 1: latitude −34.89299, longitude 114.15398, depth 1385 m;
- Channel 2: latitude −34.89848, longitude 114.13385, depth 1473 m;
- Channel 3: latitude −34.88316, longitude 114.13608, depth 1419 m.

Signal spectrograms indicate a significant amount of noise in the 0–4 Hz band. The signals in this band appear to have a broadband frequency content which is typical for AGWs^8^. We therefore filtered low frequencies (below 5 Hz) with a high pass Butterworth IIR filter.

Entropy, in information theory, is a measure of how much information is contained in a signal. Let *P*(*x*) be the probability distribution function of the discrete-time signal *x*, the entropy *H* is defined as:

20 $$H(x)=-\sum \,P({x}_{i})\,\mathrm{log}\,(P({x}_{i}))$$

Log energy entropy^19^ is defined as

21 $${H}_{L}=-\sum \,log\,(P({x}_{i}))$$

By calculating a windowed entropy value, transient signals over a noisy background can be identified^20^. This is based on the assumption that the signal randomness measure will change when the signal’s nature changes.

**Figure 8:**
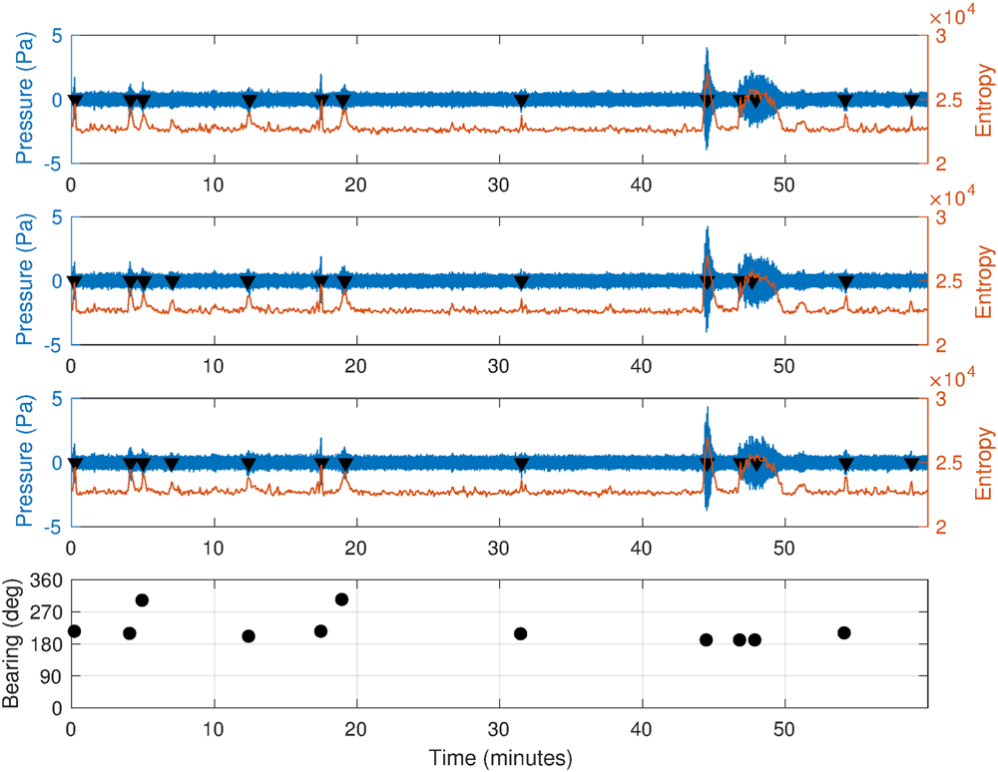
Three channels from station HA01 between 19:00 and 20:00 UTC on March 7th 2014. Windowed entropy shows peaks where a transient signal is found. These peaks (black triangle), when present on all channels, define an event. Events are then cut from their surrounding data and the bearing is calculated.

Entropy values across a window size of 4 s and a step size of 0.5 s have been calculated. As shown in Fig. 8, peaks in the entropy trace are present where transient signals are detected. A threshold of 2.3 · 10^4^ was set; all peaks which occurred within a 2 s window across all signals were considered for the subsequent bearing calculation.

After separating the signals of interest, the bearing is calculated using time of arrival based triangulation. The time of arrival is estimated based on the maximum of the cross-correlation function taken across channel pairs, which allows to obtain pairwise time of arrival differences *t*_*i*_ − *t*_*j*_ = Δ_*ij*_. All geometric parameters for the array are derived from latitude and longitude position data for the hydrophones.

**Figure 9:**
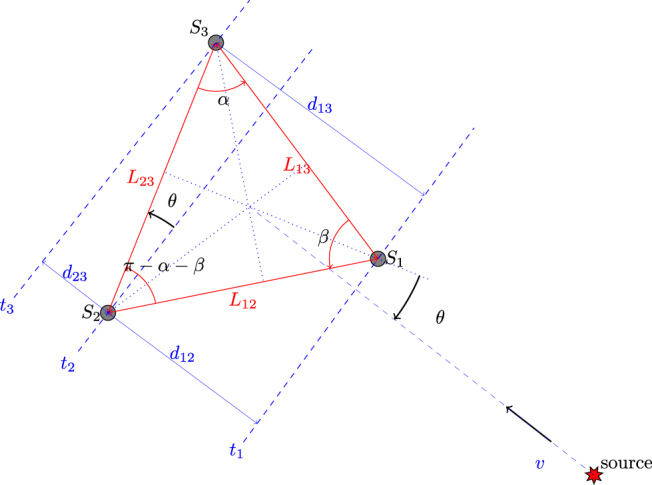
Geometry of the three hydrophones array when the hydrophones positions are known. Assuming that the wavefront radius is very large with respect to the array size, the bearing of the source can be calculated considering the pairwise arrival time differences.

Under the assumptions that wave velocity *υ* does not depend on direction and is constant through the array, the event is generated outside the array, and the event is far from the array (so that the wavefront can be considered a line), one can consider the array as shown in Fig. 9. The time difference between two sensors *t*_*i*_ − *t*_*j*_ = Δ_*ij*_ is equal to:

22 $${t}_{i}-{t}_{j}=\frac{{d}_{i}-{d}_{j}}{\upsilon }={{\rm{\Delta }}}_{ij}$$

where *d*_*i*_ is the normal distance between the i-th sensor *S*_*i*_ and the source. The distance the wavefront has to travel through between two sensors, *d*_*ij*_, can be calculated as follows:

23 $${d}_{23}={L}_{23}\,\sin \,\theta $$

24 $${d}_{12}={L}_{12}\,\sin \,(\pi -\alpha -\beta -\theta )$$

where *L*_*ij*_ is the distance between sensor *S*_*i*_ and *S*_*j*_ within the array. By rearranging Equation 24,

25 $${d}_{12}={L}_{12}[\sin \,(\alpha +\beta )\,\cos \,\theta +\,\cos \,(\alpha +\beta )\,\sin \,\theta ]$$

26 $$\frac{{d}_{12}}{{d}_{23}}=\frac{{L}_{12}}{{L}_{23}}[\sin \,(\alpha +\beta )\,\cot \,\theta +\,\cos \,(\alpha +\beta )]$$

27 $$\frac{{d}_{12}}{{d}_{23}}={A}_{1}\,\cot \,\theta +{A}_{2}$$

Regrouping, replacing *d*_*ij*_/*υ* with Δ_*ij*_ and solving for *θ*:

28 $$\theta ={\cot }^{-1}\,\{\frac{1}{{A}_{1}}[\frac{{{\rm{\Delta }}}_{21}}{{{\rm{\Delta }}}_{32}}-{A}_{2}]\}$$

29 $${A}_{1}=\frac{{L}_{12}}{{L}_{23}}\,\sin \,(\alpha +\beta )$$

30 $${A}_{2}=\frac{{L}_{12}}{{L}_{23}}\,\cos \,(\alpha +\beta )$$

The above is valid for the *S*_1_ space trisection, until Δ_21_ or Δ_31_ become zero (i.e. the source direction is parallell to one of the *S*_1_ sides). The above can be generalised by renumbering sensors based on the first hit sensor.

The additional main uncertainty in the bearing calculation is related to the time difference and sampling rate. This can be modelled as uniformly distributed between ±1/(2*f*_*s*_). On top of this, an additional normally distributed error (*μ* = 0, *σ* ^2^ = *f*_*s*_/2) can be added to account for synchronization and lag calculation uncertainties. As the bearing calculation is non-linear and is a function of the bearing itself, the simplest way to determine the accuracy is through a Monte Carlo simulation.

**Figure 10:**
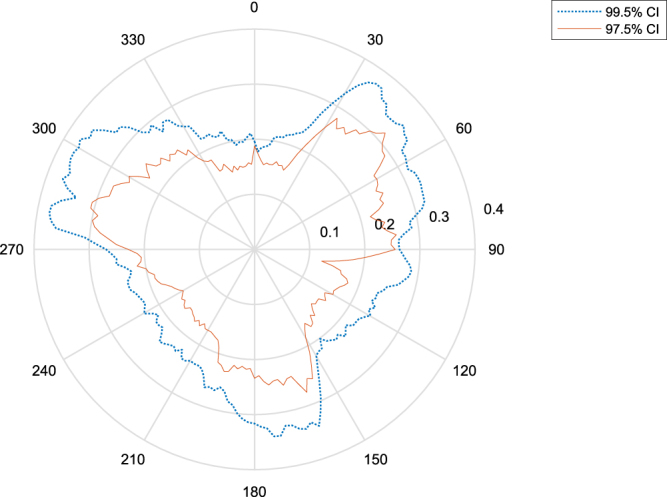
Bearing error as calculated with a Monte Carlo simulation. The graph shows that the error depends on the position along the quadrants; this is expected as the trigonometric functions used for the calculation have a large first derivative when close to *π*. The 99.5% (blue) and 97.5% (red) confidence intervals are shown on the graph.

Sources were simulated from 180 directions, uniformly separated by 2 degrees. From the exact times of arrival, the above mentioned normally distributed error was superimposed. *N* = 10^6^ samples were extracted for each direction. After this, times were rounded to the nearest 1/*f*_*s*_ to simulate the effect of discrete sampling. Results for 99.5% and 97.5% percentiles of the Monte Carlo simulation are shown in Fig. 10. For this reason, taking into account time measurement errors, the bearing calculations are considered to be accurate to ±0.4° using a a 99.5% confidence interval.

### Data Availability

Data can be accessed by direct inquiries to kadriu@cardiff.ac.uk.
